# Supplementary material for: Automated analysis of fibrous cap in intravascular optical coherence tomography images of coronary arteries
Source: Sci Rep. 2022 Dec 12;12:21454. doi: 10.1038/s41598-022-24884-1 (PMC9744742; doi:10.1038/s41598-022-24884-1)
Supplement: Supplementary file 1 — Supplementary Information. [file 41598_2022_24884_MOESM1_ESM.pdf]

## Supplementary figures

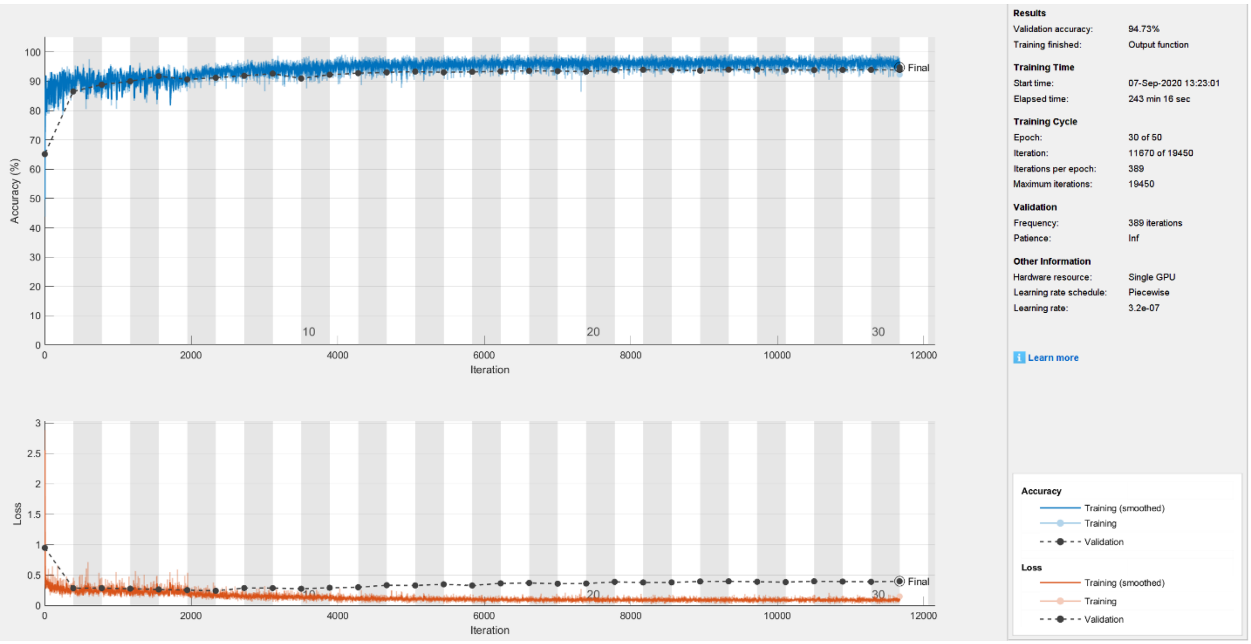


Fig. S1. Learning curves of training and validation for lipid segmentation obtained from one cross-validation round. Blue is the training curve, and orange is the loss curve. The black dotted lines are (top) validation accuracy and (bottom) validation loss. The curves are smoothened for better visualization.

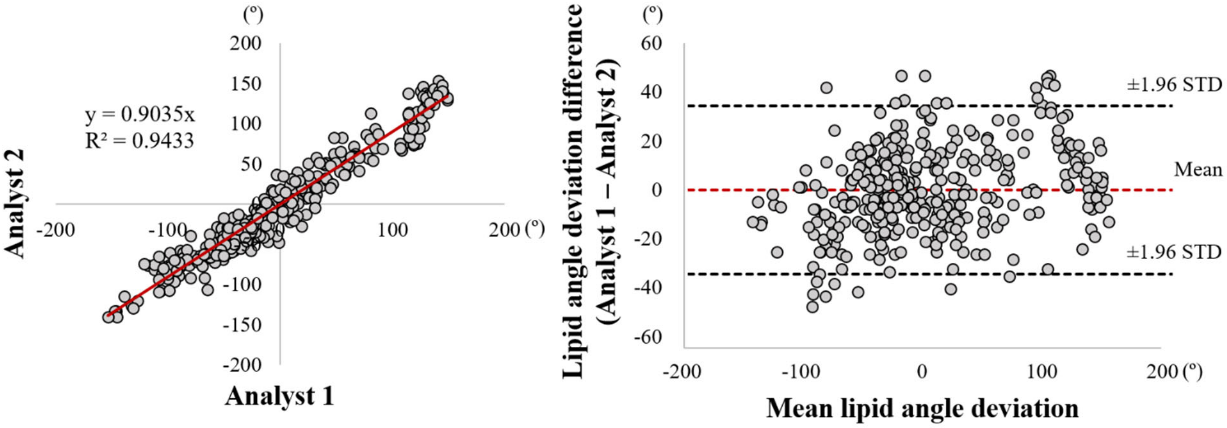


Fig. S2. Inter-observer variability test of lipid angle deviation between two analysts after automated prediction + manual editing. Linear regression plot (left) and Bland-Altman plot (right) are shown. The R-squared ($R^2$) value was 0.943 (left), and the mean bias between the two analysts was 0.0°±17.3° (right).

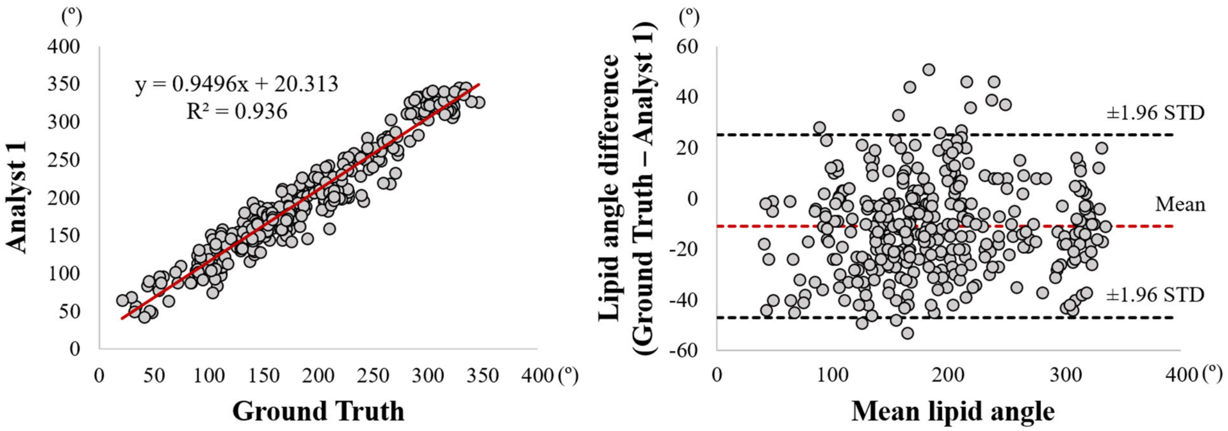


Fig. S3. Comparison of lipid angle between ground truth and automated prediction + manual editing. Panels are (left) linear regression plot and (right) Bland-Altman plot. The R-squared ($R^2$) value was 0.936 (left), and the mean bias was -11.0°±18.4° (right).
